# Complete Genome Sequence of Citrobacter freundii Myophage Maleficent

**DOI:** 10.1128/MRA.01153-19

**Published:** 2019-10-17

**Authors:** Hayden H. Wright, Victoria Berkowitz, Chandler O’Leary, Heather Newkirk, Rohit Kongari, Jason Gill, Mei Liu

**Affiliations:** a Center for Phage Technology, Texas A&M University, College Station, Texas, USA; Queens College

## Abstract

Citrobacter freundii, a member of the *Enterobacteriaceae* family, has been linked to opportunistic infections in neonates and immunocompromised adults. Here, we report the complete genome sequence of a T4-like myophage, Maleficent, which infects C. freundii.

## ANNOUNCEMENT

Citrobacter freundii is a Gram-negative bacterium belonging to the family *Enterobacteriaceae*. Apart from causing opportunistic nosocomial urinary tract infections in immunocompromised patients (1, 2), C. freundii has been associated with fatal cases of neonatal meningitis (3, 4). With the rise of antibiotic-resistant Citrobacter strains (5), alternative treatment options such as phage therapy are being explored (6, 7). The isolation and characterization of bacteriophages infecting C. freundii, such as the myophage Maleficent described in this study, could help with such strategies.

Phage Maleficent was isolated using a C. freundii strain from a municipal wastewater sample collected from College Station, TX, in 2015. LB broth or agar (Difco) was used to culture the host bacteria and for phage enrichment at 37°C with aeration. Phage isolation and propagation were conducted using the soft-agar overlay method (8). Maleficent was identified as a myophage using negative-stain transmission electron microscopy performed at the Texas A&M University Microscopy and Imaging Center, as described previously (9). Phage genomic DNA was prepared using a modified Promega Wizard DNA cleanup kit protocol (9). Pooled indexed DNA libraries were prepared using the Illumina TruSeq Nano LT kit, and the sequence was obtained from the Illumina MiSeq platform using the MiSeq V2 500-cycle reagent kit, following the manufacturer’s instructions, producing 773,101 paired-end reads for the index containing the phage Maleficent genome. FastQC 0.11.5 (https://www.bioinformatics.babraham.ac.uk/projects/fastqc/) was used to quality control the reads. The reads were trimmed with the FASTX-Toolkit 0.0.14 (http://hannonlab.cshl.edu/fastx_toolkit/download.html) before being assembled using SPAdes 3.5.0 (10). Contig completion was confirmed by PCR using primers (5′-AACCGTTTAGTAACCCTGTTAG-3′ and 5′-ACATGTACAACCTGCATCAC-3′) facing off the ends of the assembled contig and Sanger sequencing of the resulting product, with the contig sequence manually corrected to match the resulting Sanger sequencing read. GLIMMER 3.0 (11) and MetaGeneAnnotator 1.0 (12) were used to predict protein-coding genes with manual verification, and tRNA genes were predicted with ARAGORN 2.36 (13). Rho-independent termination sites were identified via TransTerm (http://transterm.cbcb.umd.edu/). Sequence similarity searches were done by BLASTp 2.2.28 (14) against the NCBI nr, UniProt Swiss-Prot (15), and TrEMBL databases. InterProScan 5.15–54.0 (16), LipoP (17), and TMHMM v2.0 (18) were used to predict protein function. All analyses were conducted at default settings via the CPT Galaxy (19) and Web Apollo (20) interfaces (https://cpt.tamu.edu/galaxy-pub).

Myophage Maleficent has an 89,570-bp-long genome (assembled at 34.7-fold coverage) with 34.7% GC content, which is lower than that of the host (51.6%) (21). Overall, 137 protein-coding sequences were annotated, leading to a coding density of 81%. About 76% of the annotated proteins in the Maleficent genome have homologs in phage T4 (NCBI RefSeq accession no. NC_000866). Most of the genes annotated with a function were either linked to DNA replication (such as polynucleotide kinase, terminase large subunit, DNA ligase, DNA polymerase, DNA helicase, thymidylate synthase, and exonuclease) or involved in virion morphogenesis (such as head maturation protease, major capsid protein, tail protein, tape measure protein, baseplate assembly protein, and tail fiber protein). Genes associated with host lysis, such as class III holins, lysozymes, and an overlapping spanin pair, were also annotated in the genome.

### Data availability.

The genome sequence of phage Maleficent was submitted to GenBank as accession no. MH920362. The associated BioProject, SRA, and BioSample accession numbers are PRJNA222858, SRR8556430, and SAMN10909361, respectively.
